# Prognosis prediction model based on competing endogenous RNAs for recurrence of colon adenocarcinoma

**DOI:** 10.1186/s12885-020-07163-y

**Published:** 2020-10-07

**Authors:** Li Peng Jin, Tao Liu, Fan Qi Meng, Jian Dong Tai

**Affiliations:** Department of Colorectal & Anal Surgery, First Hospital Bethune of Jilin University, No. 71, Xinmin Street, Chaoyang District, Changchun, 130000 Jilin China

**Keywords:** Colon adenocarcinoma, Recurrence, Prognosis prediction model, ceRNAs

## Abstract

**Background:**

Colon adenocarcinoma (COAD) patients who develop recurrence have poor prognosis. Our study aimed to establish effective prognosis prediction model based on competing endogenous RNAs (ceRNAs) for recurrence of COAD.

**Methods:**

COAD expression profilings downloaded from The Cancer Genome Atlas (TCGA) were used as training dataset, and expression profilings of GSE29623 retrieved from Gene Expression Omnibus (GEO) were set as validation dataset. Differentially expressed RNAs (DERs) between non-recurrent and recurrent specimens in training dataset were screened, and optimum prognostic signature DERs were revealed to establish prognostic score (PS) model. Kaplan-Meier survival analysis was conducted for PS model, and GEO dataset was used for validation. Prognosis prediction efficiencies were evaluated by area under curve (AUC) and C-index. Meanwhile, ceRNA regulatory network was constructed by using signature mRNAs, lncRNAs and miRNAs.

**Results:**

We identified 562 DERs including 42 lncRNAs, 36 miRNAs, and 484 mRNAs. PS prediction model, consisting of 17 optimum prognostic signature DERs, showed that high risk group had significantly poorer prognosis (5-year AUC = 0.951, C-index = 0.788), which also validated in GSE29623. Prognosis prediction model incorporating multi-RNAs with pathologic distant metastasis (M) and pathologic primary tumor (T) (5-year AUC = 0.969, C-index = 0.812) had better efficiency than clinical prognosis prediction model (5-year AUC = 0.712, C-index = 0.680). In the constructed ceRNA regulatory network, lncRNA *NCBP2-AS1* could interact with *hsa-miR-34c* and *hsa-miR-363*, and lncRNA *LINC00115* could interact with *hsa-miR-363* and *hsa-miR-4709*. *SIX4*, *GRAP*, *NKAIN4*, *MMAA*, and *ERVMER34–1* are regulated by *hsa-miR-4709*.

**Conclusion:**

Prognosis prediction model incorporating multi-RNAs with pathologic M and pathologic T may have great value in COAD prognosis prediction.

## Background

Colon cancer is the first leading cancer type in digestive system, and approximately 101,420 new cases of colon cancer will be diagnosed in United States in 2019 with the estimated deaths of 51,020 [1]. Colon adenocarcinoma (COAD) counts for more than 80% of all colon cancers, and the remaining small proportion of colon cancers are related to sarcomas and squamous cell carcinomas [2, 3]. Retrospectively study conducted by Cass et al. has revealed that 37% patients developed local recurrence and distant metastases after complete primary resection, and local recurrence without clinical evidence of distant metastases was the most common cause of death within 5 years [4]. Even though survival of patients with colon cancer has been improved along with the developing of new chemotherapeutics and advanced techniques, the prognosis of patients developed recurrence still remains poor and early recurrence have the possibility to be cured by salvage surgery [5, 6]. Thus, it is urgently to find the prognostic markers and construct effective prognosis prediction model for the recurrence of COAD.

It is widely acknowledged that competing endogenous RNAs (ceRNAs) could interact with mRNAs by competing with miRNAs, and miRNA-mediated interactions between long non-coding RNAs (lncRNAs) and mRNAs exist in the progressing of various diseases [7–9]. The lncRNA *H19* has been reported to function as a ceRNA for *miR-138* and *miR-200a* to suppress the target gene expressions of *Vimentin*, *ZEB1*, and *ZEB2*, thereafter to promote epithelial to mesenchymal transition in colorectal cancer [10]. Moreover, lncRNA *H19* could also up-regulate cancer-related mRNA expressions by functioning as a ceRNA to influence phosphatidylinositol-3-kinase (PI3K)/Akt signaling pathway, and lncRNA *H19* is associated with poor prognosis in colorectal cancer [11]. The lncRNA *ATB* could inhibit the expression of E-cadherin, thus contributing to the colon cancer progression via epithelial to mesenchymal transition, and higher levels of lncRNA *ATB* are related to poor prognosis [12]. Recently, the ceRNA regulatory networks of lncRNAs, miRNAs and mRNAs have been also constructed in many studies to reveal the molecular mechanisms involved in initiation and progression of human colon adenocarcinoma [13–15]. However, there are few studies about ceRNAs based prediction models of COAD recurrence. In the study of Chen et al., they established ceRNA network for differentially expressed RNAs (DERs) between 15 non-recurrent and 98 recurrent COAD patients from The Cancer Genome Atlas (TCGA) database [16]. Meanwhile, Chen et al. identified the prognostic lncRNA markers by using multivariate regression and established a nomogram for recurrence prediction based on the identified lncRNA biomarkers and clinical covariates [16].

In order to further reveal the recurrence prediction markers, the expression profilings of 367 COAD samples (279 non-recurrent and 88 recurrent ones) downloaded from TCGA were used as training dataset, and expression profilings of 65 COAD samples from Gene Expression Omnibus (GEO) were considered as validation dataset. Optimum prognostic signature DERs were identified via the L1 penalized lasso estimation in addition to multivariate regression analysis. Prognosis prediction efficiency of the ceRNAs-based prognostic score prediction model was compared with that of clinical prognostic prediction model.

## Materials and methods

### Data source and preprocessing

The mRNA, lncRNA and miRNA sequencing data of COAD samples based on the platform of Illumina HiSeq 2000 RNA Sequencing were downloaded from TCGA (https://portal.gdc.cancer.gov/projects/TCGA-COAD). The mRNA along with lncRNA expression profilings (RNA-seq data) of 512 COAD samples and miRNA expression profilings (miRNA-seq data) of 461 COAD samples were downloaded from TCGA A total of 367 COAD samples that had both of mRNA expression profilings and miRNA expression profilings were obtained by mapping the clinical data for each sample. These expression profilings of the 367 COAD samples, including 279 non-recurrent and 88 recurrent specimens, were used as training dataset.

Meanwhile, RNA expression profilings of human colon adenocarcinoma were retrieved by searching the keywords of “colon adenocarcinoma” and “*Homo sapiens*” from National Center for Biotechnology Information (NCBI) GEO (https://www.ncbi.nlm.nih.gov/geo/) for validation. The datasets were retained when meet the following criteria: expression profilings of tumor tissue samples from COAD patients; containing lncRNA, mRNA and miRNA expression profilings; with corresponding available clinical information about recurrence and prognosis. Subsequently, GSE29623 [17] consisted of lncRNA, mRNA and miRNA expression profilings of 65 colon adenocarcinomas based on the platforms of GPL570 [HG-U133_Plus_2] Affymetrix Human Genome U133 Plus 2.0 Array and GPL11162 NIH Taqman Human MicroRNA Array v.2 (https://www.ncbi.nlm.nih.gov/geo/query/acc.cgi?acc=GSE29623) was used as validation dataset.

### Identification of differentially expressed RNAs

The lncRNAs, mRNAs and miRNAs in training and validation datasets were annotated by using Human Genome Organisation (HUGO) Gene Nomenclature Committee (HGNC, http://www.genenames.org/) [18]. Afterwards, the differentially expressed RNAs between non-recurrent and recurrent specimens in training dataset were screened by Limma (Linear Models for Microarray Data) package (Version 3.34.7; https://bioconductor.org/packages/release/bioc/html/limma.html) [19] of R3.4.1. The cut-off criteria were set as false discovery rate (FDR) < 0.05 and |log_2_ fold change (FC) | > 0.5. Hierarchical clustering analysis based on centered Pearson correlation was performed for all the identified DERs via the pheatmap (Version 1.0.8, https://cran.r-project.org/web/packages/pheatmap/index.html) of R3.4.1 [20, 21].

### Construction and validation of prognosis prediction model based on prognostic score

The DERs related to prognosis were firstly uncovered by using the univariate Cox regression analysis in Survival package (Version2.41–1, http://bioconductor.org/packages/survivalr/) [22]. Then, multivariate Cox regression analysis was conducted to screen the independent prognostic DERs with the threshold of log-rank *p* value < 0.05. Thereafter, the optimum prognostic signature DERs were identified via the L1 penalized lasso estimation based Cox-Proportional Hazards (Cox-PH) model in penalized package (Version0.9–50, http://bioconductor.org/packages/penalized/) of R3.4.1. When the maximal cross-validation likelihood run 1000 times, the optimal lambda was determined. The prognostic score was calculated based on the identified optimum prognostic signature DERs according to the following formula:

$$ \mathrm{Prognostic}\ \mathrm{score}\ \left(\mathrm{PS}\right)=\sum {\upbeta}_{\mathrm{DERs}}\times {\mathrm{Exp}}_{\mathrm{DERs}} $$

Where β _DERs_ referred to the LASSO coefficients of signature DERs, and Exp _DERs_ denoted the expression levels of signature DERs.

The PS value of each sample in training dataset was calculated, and samples were divided into high risk group (PS > median value) and low risk group (PS < median value) by using median value as the cut-off point. The Kaplan-Meier curves were plotted by survival package (Version2.41–1) of R3.4.1 [22]. At the same time, the PS value of each sample in validation dataset was also calculated using the expression levels, and samples were divided into high risk group and low risk group with median value as the cut-off point. The Kaplan-Meier curves for samples in validation dataset were plotted by survival package (Version2.41–1) of R3.4.1 [22].

### Screening independent prognostic clinical factors and prognosis prediction efficiencies

The independent prognostic clinical factors for training dataset were screened by using the univariate and multivariate Cox regression analyses in Survival package (Version2.41–1, http://bioconductor.org/packages/survivalr/) [22] with the threshold of log-rank *p* value < 0.05. Afterwards, the clinical prognostic prediction model was constructed based on the screened independent prognostic clinical factors.

The prognosis prediction efficiencies of clinical prognostic prediction model and the PS prediction model were evaluated by pROC package (Version 1.14.0, https://cran.r-project.org/web/packages/pROC/index.html) of R3.4.1 [23] to calculate the area under ROC (AUC; AUC = 0.5: no discriminatory power, AUC = 1: perfect discriminatory power) and by survcomp (Version 1.34.0, http://www.bioconductor.org/packages/release/bioc/html/survcomp.html) of R3.4.1 [24, 25] to calculate the C-index (C-index > 0.7: acceptable discriminatory power; C-index > 0.8: excellent discriminatory power; C-index > 0.9: outstanding discriminatory power).

### ceRNA regulatory network

The regulatory interactions between signature lncRNAs and signature miRNAs identified above were retrieved from the database of DIANA-LncBase V2 (http://carolina.imis.athena-innCOADation.gr/diana_tools/web/index.php?r=lncbasev 2%2Findex-experimental). Cytoscape (Version 3.6.1, https://cytoscape.org/) [26] was used to visualize the lncRNA-miRNA regulatory network.

The target genes of differentially expressed miRNAs were predicted based on the starBase (Version 2.0, http://starbase.sysu.edu.cn/) [27], which contains prediction data from five databases including targetScan, picTar, RNA22, PITA and miRanda. The regulatory interactions predicted in at least one of the five databases were remained. Signature mRNAs identified above were mapped into the predicted target genes to construct the signature miRNA-mRNA regulatory network, which was also visualized by Cytoscape (Version 3.6.1, https://cytoscape.org/) [26]. Subsequently, lncRNA-miRNA regulatory network was integrated with signature miRNA-mRNA regulatory network to construct the ceRNA regulatory network by Cytoscape (Version 3.6.1, https://cytoscape.org/) [26].

## Results

### Screened DERs between non-recurrent and recurrent COAD samples

**Fig. 1:**
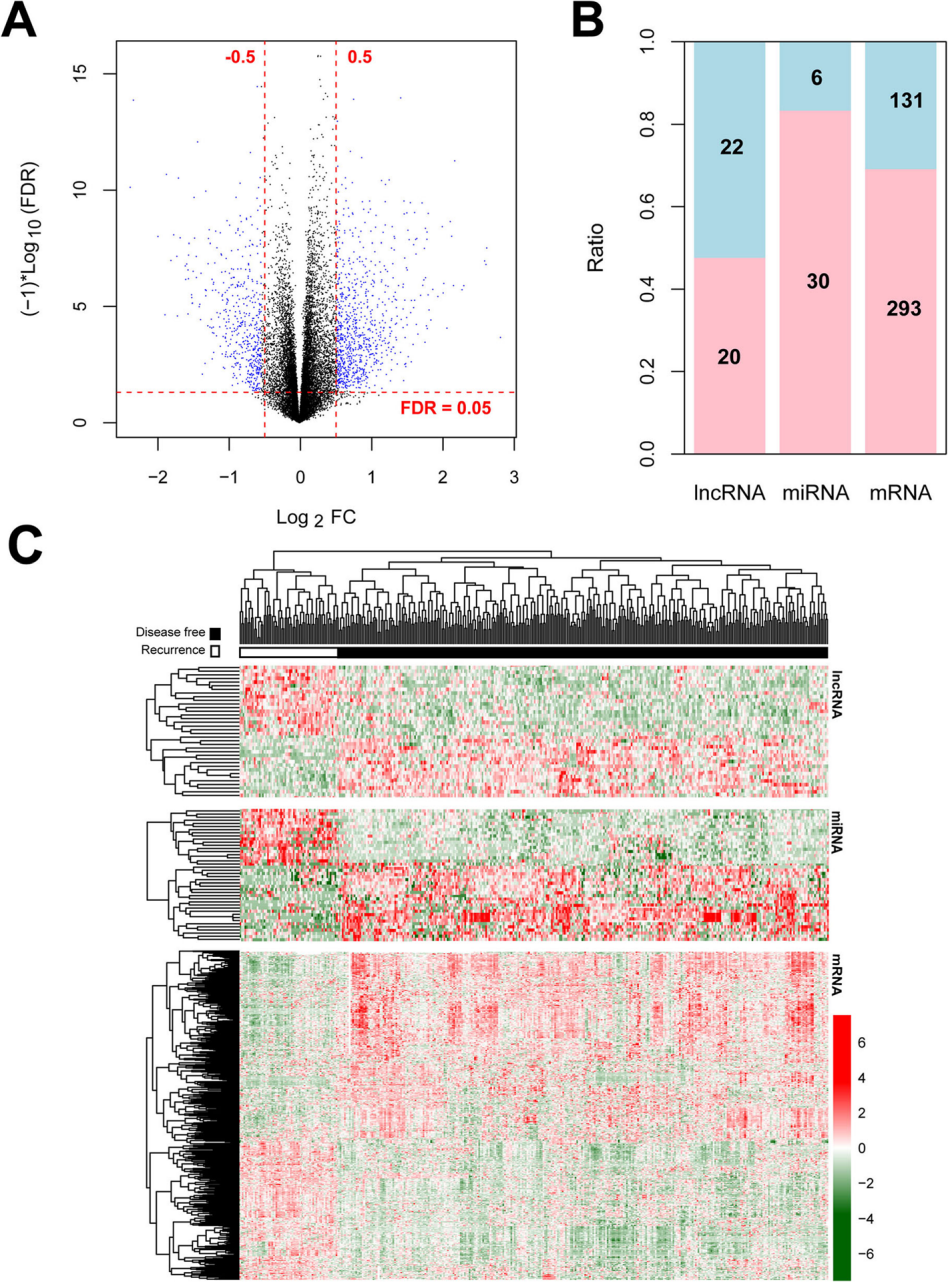
Identification and hierarchical clustering analysis of differentially expressed RNAs (DERs) between 279 non-recurrent and 88 recurrent colon adenocarcinoma (COAD) samples in training dataset. A, the identified DERs with the thresholds of false discovery rate (FDR) < 0.05 and |log_2_ fold change (FC) | > 0.5. Blue dots represent DERs. B, the ratios of up-regulated and down-regulated lncRNAs, miRNAs and mRNAs. Pink column and blue column represent up-regulated and down-regulated DERs, respectively. C, the hierarchical clustering analysis of differentially expressed lncRNAs, miRNAs or mRNAs. Obvious color contrast was observed suggesting DERs could well distinguish the non-recurrent COAD samples from recurrent COAD samples

After annotation, 13,834 mRNAs, 539 lncRNAs and 540 miRNAs were obtained in training and validation datasets. In total, 562 DERs were identified by Limma between 279 non-recurrent and 88 recurrent COAD samples in the TCGA training dataset, including 42 lncRNAs (20 up-regulated and 22 down-regulated ones), 484 mRNAs (393 up-regulated and 131 down-regulated ones), and 36 miRNAs (30 up-regulated and 6 down-regulated ones) (Fig. 1a, b). Meanwhile, the hierarchical clustering analysis results showed the expression levels of differentially expressed lncRNAs, miRNAs or mRNAs could well distinguish the non-recurrent COAD samples from recurrent COAD samples (Fig. 1b).

### Prognostic score prediction model

There were 227 DERs (204 mRNAs, 11 lncRNAs and 12 miRNAs) related to prognosis according to the univariate Cox regression analysis in Survival package with the log-rank *p* value < 0.05. Furthermore, 48 independent prognostic DERs (4 lncRNAs, 6 miRNAs and 38 mRNAs) were identified via the multivariate Cox regression analysis in Survival package. The L1 penalized estimation based Cox-PH model in penalized package revealed 17 optimum prognostic signature DERs (6 miRNAs, 2 lncRNAs and 9 mRNAs), including 3 protective RNAs (*hsa-mir-105-1*, *hsa-mir-3609*, and *MMAA*) and 14 risky RNAs (*LINC00115*, *NCBP2-AS1*, *hsa-mir-34c*, *hsa-mir-363*, *hsa-mir-4709*, *hsa-mir-891a*, *ALOX15*, *ERVMER34–1*, *GRAP*, *KCP*, *NKAIN4*, *PADI1*, *SIX4*, and *SLC16A8*) based on the hazard ratio (HR) by multivariate Cox regression analysis (Table 1).

**Table 1:** The optimum prognostic signature DERs identified by L1 penalized estimation based Cox-Proportional Hazards (Cox-PH) model

Symbol	Chromosome location	Type	Multi-variate Cox regression analysis			Lasso coefficient
			HR	95%CI	***P*** value	
**Protective RNAs**						
*hsa-mir-105-1*	Xq28	miRNA	0.629	0.444–0.891	4.990E-02	−0.034
*hsa-mir-3609*	7q22.1	miRNA	0.635	0.486–0.831	9.340E-04	−0.394
MMAA	4q31.21	mRNA	0.680	0.475–0.974	3.520E-02	−0.781
**Risky RNAs**						
*LINC00115*	1p36.33	lncRNA	1.692	1.103–2.595	1.602E-02	0.238
*NCBP2-AS1*	3q29	lncRNA	2.121	1.450–3.104	1.080E-04	0.122
*hsa-mir-34c*	11q23.1	miRNA	1.150	1.013–1.304	3.019E-02	0.039
*hsa-mir-363*	Xq26.2	miRNA	1.250	1.121–1.395	6.490E-05	0.243
*hsa-mir-4709*	14q24.3	miRNA	1.480	1.200–1.825	2.510E-04	0.635
*hsa-mir-891a*	Xq27.3	miRNA	1.165	1.028–1.320	1.695E-02	0.196
*ALOX15*	17p13.2	mRNA	1.495	1.229–1.818	5.660E-05	0.395
*ERVMER34–1*	4q12	mRNA	1.259	1.058–1.497	9.378E-03	0.305
*GRAP*	17p11.2	mRNA	1.782	1.242–2.556	1.704E-03	0.542
*KCP*	7q32.1	mRNA	1.258	1.158–1.652	9.050E-03	0.231
*NKAIN4*	20q13.33	mRNA	2.221	1.396–3.533	7.510E-04	0.220
*PADI1*	1p36.13	mRNA	1.326	1.159–1.517	3.970E-05	0.373
*SIX4*	14q23.1	mRNA	1.543	1.026–2.319	3.713E-02	0.423
*SLC16A8*	22q13.1	mRNA	2.161	1.303–3.583	2.821E-03	0.881

*HR* hazard ratio, *CI* confidence interval

**Fig. 2:**
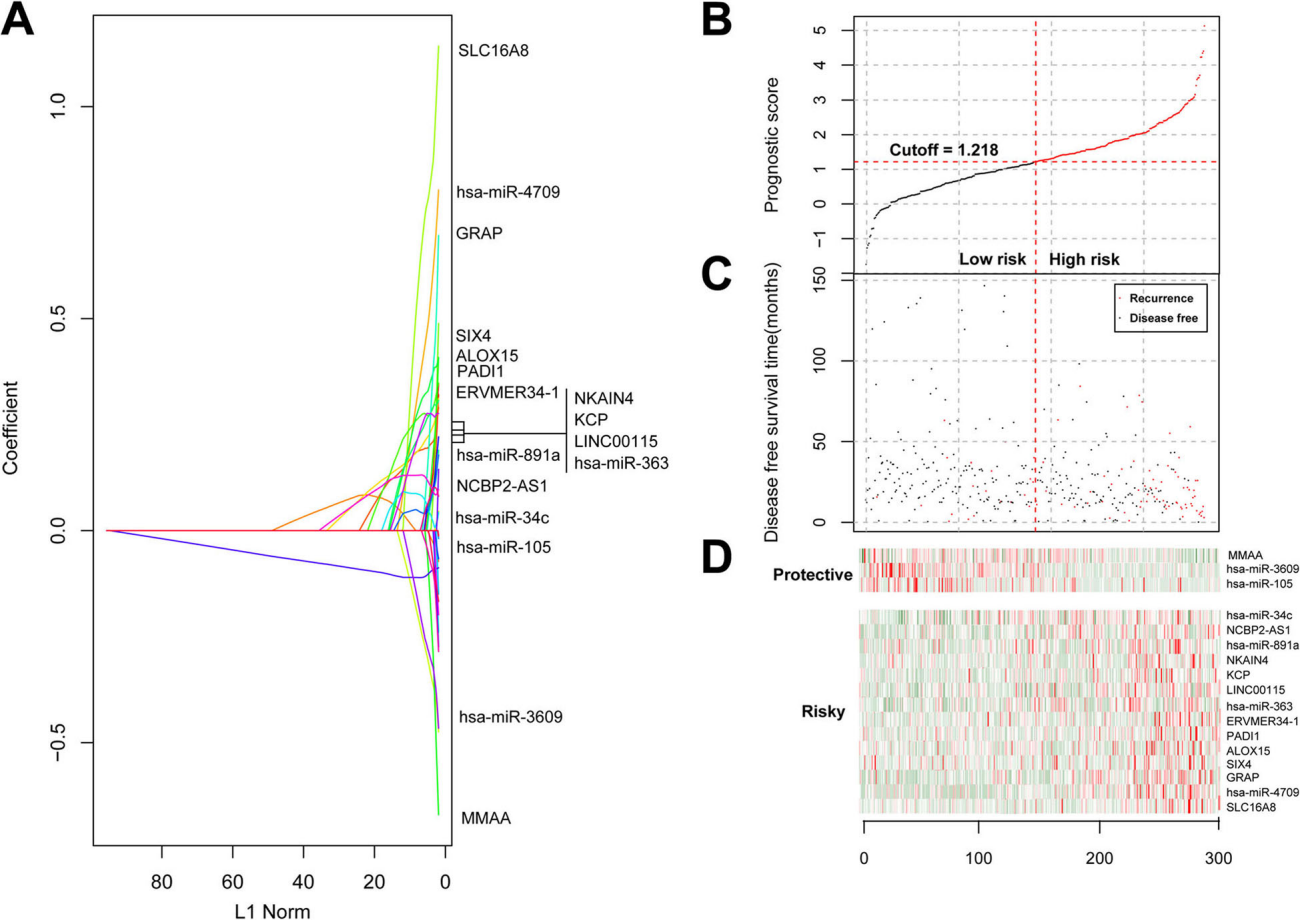
The 17 optimum prognostic signature DERs. A, the lasso coefficient curves of the 17 signature DERs. B, calculated prognostic score (PS) of each sample in training dataset. C, prognosis of patients in training dataset between high risk group and low risk group. D, expression levels of 3 protective RNAs and 14 risky RNAs of samples in high risk group and low risk group

The PS value of each sample in training dataset was calculated based on the LASSO coefficients (Fig. 2a) and expression levels of the 17 signature DERs. Samples were divided into high risk group and low risk group with the median value of 1.218 as the cut-off point (Fig. 2b). The survival time and recurrent status of patients in high risk group and low risk group showed that patients in low risk group had obviously longer survival time and lower proportion of recurrence (Fig. 2c). Meanwhile, expression levels of signature DERs in each sample revealed that the 3 protective RNAs were up-regulated in low risk group, while the other 14 risky RNAs were up-regulated in high risk group (Fig. 2d).

**Fig. 3:**
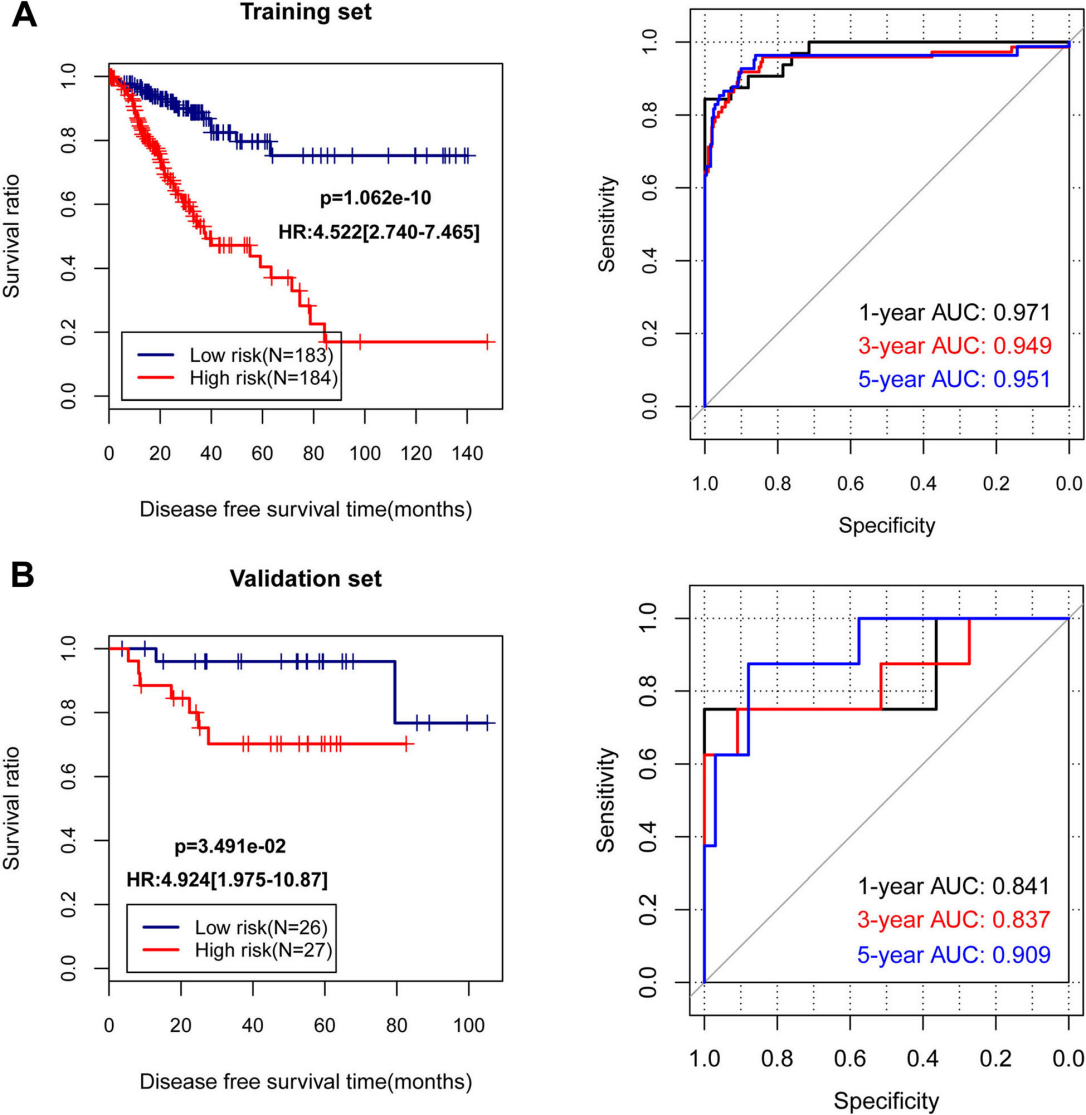
The Kaplan-Meier curves and receiver operating characteristic curves for training and validation datasets. A, the Kaplan-Meier curves of high risk group and low risk group and receiver operating characteristic (ROC) curves for training dataset. B, the Kaplan-Meier curves of high risk group and low risk group and receiver operating characteristic curves for validation dataset

The Kaplan-Meier curves of training dataset showed high risk group and low risk group classified by PS prediction model had significantly different prognosis (*p* = 1.062e-10) (Fig. 3a). The AUC of 1-year, 3-year, and 5-year ROC of training dataset was 0.971, 0.949, and 0.951, respectively. With regarding to the validation dataset, high risk group also showed significantly lower survival ration in comparison with the low risk group (*p* = 3.491e-02) (Fig. 3b). Meanwhile, the AUC of 1-year, 3-year, and 5-year ROC of validation dataset was 0.841, 0.837, and 0.909, respectively.

### Independent prognostic clinical factors and prognosis prediction efficiencies of different models

**Fig. 4:**
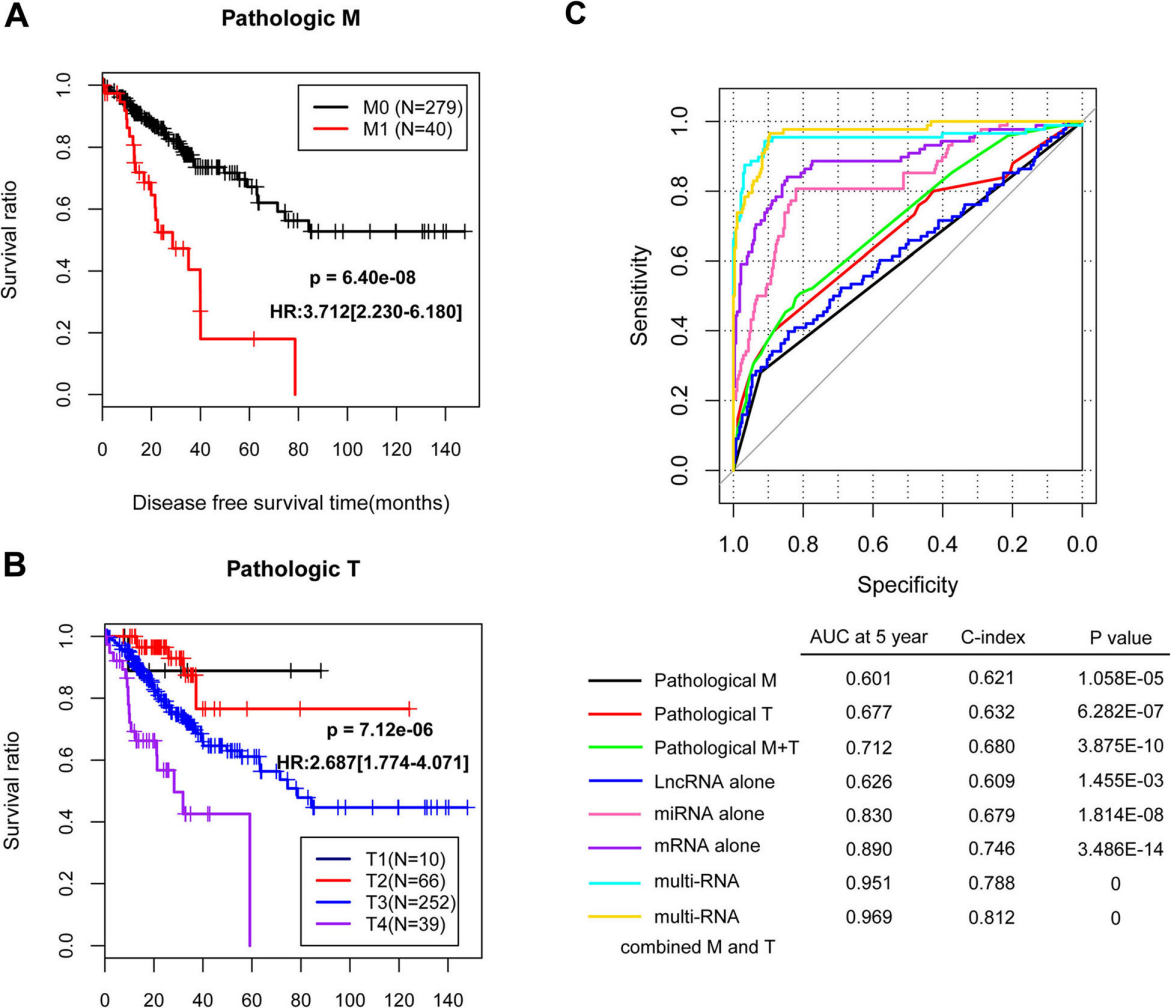
The Kaplan-Meier curves of pathologic distant metastasis (M) and pathologic primary tumor (T) in training dataset, and prediction efficiencies of different prognosis prediction models. A, Kaplan-Meier curves of pathologic M0 and M1. B, Kaplan-Meier curves of pathologic T1, T2, T3, and T4. C, ROC curves of different prognosis prediction models

Three independent prognostic clinical factors, i.e. pathologic distant metastasis (M) (*p* = 2.65E-02), pathologic primary tumor (*p* = 2.23E-02), and PS model status (*p* = 1.29E-05), were found according to univariate and multivariate Cox regression analyses (Table 2). Moreover, the Kaplan-Meier curves of pathologic M and pathologic T showed low pathologic M or pathologic T stage had significantly higher survival ratio (Fig. 4a, b). The prognosis prediction model constructed by both of pathologic M and pathologic T had better prognosis prediction efficiency (5-year AUC = 0.712, C-index = 0.680) than prediction model of pathologic M or pathologic T alone. The prognosis prediction model constructed by mRNAs (C-index = 0.746), or multi-RNAs (C-index = 0.788) had a C-index value larger than 0.7, suggesting acceptable discriminatory power. Moreover, prognosis prediction model constructed by multi-RNAs combined pathologic M and pathologic T had a 5-year AUC of 0.969 and a C-index of 0.812, suggesting excellent discriminatory power (Fig. 4c).

**Table 2:** The independent prognostic clinical factors according to univariate and multivariate Cox regression analyses

Clinical characteristics	TCGA (***N*** = 367)	Uni-variables cox			Multi-variables cox		
		HR	95%CI	P	HR	95%CI	P
Age (years, mean ± SD)	65.40 ± 13.21	0.989	0.973–1.006	2.26E-01	–	–	–
Gender (Male/Female)	198/169	1.485	0.967–2.283	6.93E-02	–	–	–
Pathologic M (M0/M1/−)	279/40/48	3.712	2.230–6.180	6.40E-08	3.786	1.168–12.265	2.65E-02
Pathologic N (N0/N1/N2)	223/90/54	2.053	1.588–2.654	1.07E-08	1.404	0.755–2.609	2.83E-01
Pathologic T (T1/T2/T3/T4)	10/66/252/39	2.687	1.774–4.071	7.12E-06	2.159	1.116–4.176	2.23E-02
Pathologic stage (I / II / III / IV)	66/144/108/40	1.982	1.550–2.535	2.45E-08	0.733	0.313–1.716	4.75E-01
Lymphovascular invasion (Yes/No/−)	121/213/33	1.968	1.266–3.059	2.19E-03	0.709	0.357–1.407	3.25E-01
Vascular invasion (Yes/No/−)	68/250/49	2.211	1.361–3.589	1.00E-03	0.983	0.464–2.079	9.63E-01
PS model status (High/ Low)	183/184	4.522	2.740–7.465	1.06E-10	4.053	2.161–7.601	1.29E-05
Tumor status (Recurrence/Free)	88/279	–	–	–	–	–	–
Disease free survival time (months, mean ± SD)	28.30 ± 25.41	–	–	–	–	–	–

*TCGA* The Cancer Genome Atlas, *HR* hazard ratio, *CI* confidence interval, *SD* standard deviation

### ceRNA regulatory network

**Fig. 5:**
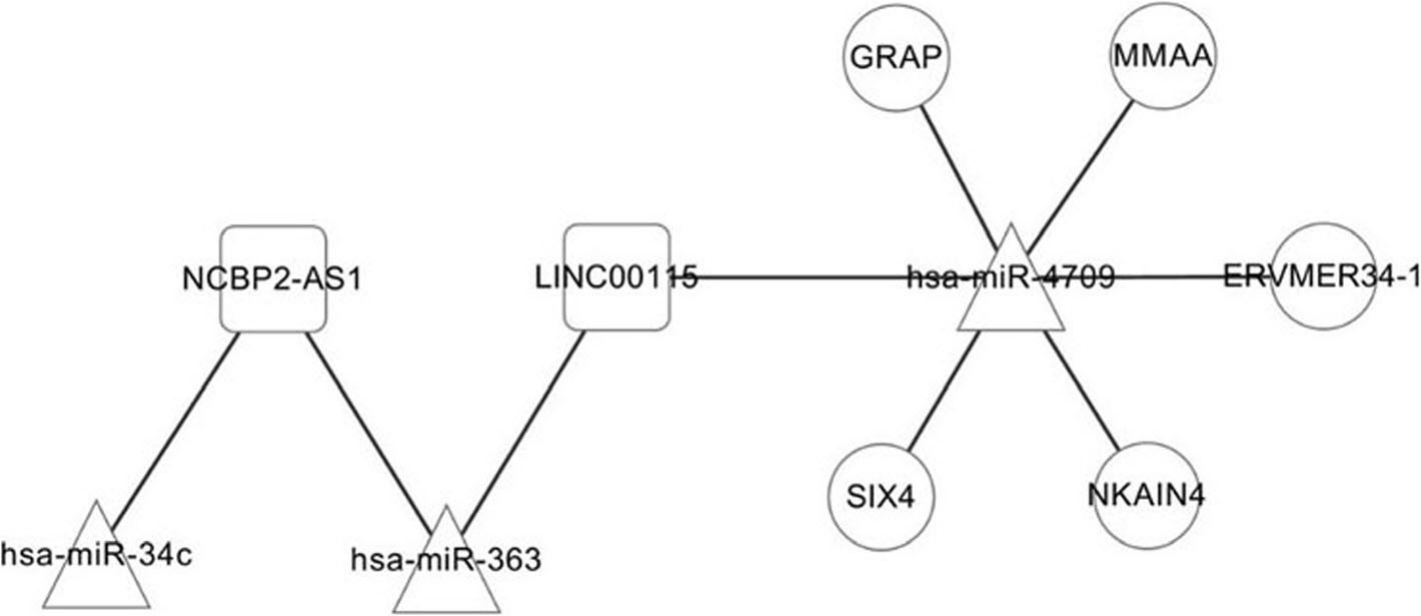
The constructed competing endogenous RNA (ceRNA) regulatory network. Square, triangle and circle nodes represent lncRNAs, miRNAs, and mRNAs, respectively

Based on the DIANA-LncBasev2 database, the regulatory interactions between signature lncRNAs and miRNAs were obtained. The lncRNA *NCBP2-AS1* could interact with *hsa-miR-34c* and *hsa-miR-363*, and lncRNA *LINC00115* could interact with *hsa-miR-363* and *hsa-miR-4709*. The target genes of signature miRNAs were predicted by starBase and regulatory interactions involving signature mRNAs were remained. *SIX4*, *GRAP*, *NKAIN4*, *MMAA*, and *ERVMER34–1* are regulated by *hsa-miR-4709*. All these regulatory interactions were used for ceRNA regulatory network construction. Three miRNAs (*hsa-miR-34c*, *hsa-miR-363* and *hsa-miR-4709*), two lncRNAs (*NCBP2-AS1* and *LINC00115*), and five mRNAs (*SIX4*, *GRAP*, *NKAIN4*, *MMAA*, and *ERVMER34–1*) were included in the ceRNA regulatory network (Fig. 5).

## Discussion

Colon adenocarcinoma is one of the most common causes of death in adults, causing a major public health problem over the world [1]. The current strategies for treating colon cancer are mainly radical surgery and chemotherapy, while the prognosis of colon cancer patients who develop distant metastasis and local recurrence after treatments would become worse [28, 29]. Therefore, effective novel biomarkers and reliable prognosis prediction model for COAD recurrence are of great help in selecting treatment strategies for patients according to the recurrence risk. In our study, 562 DERs (42 lncRNAs, 36 miRNAs, and 484 mRNAs) were identified between 279 non-recurrent and 88 recurrent COAD sample profilings downloaded from the TCGA. According to the univariate Cox regression analysis, multivariate Cox regression analysis, and L1 penalized estimation based Cox-PH model, 17 optimum prognostic signature DERs were revealed, including 3 protective RNAs (*hsa-mir-105-1*, *hsa-mir-3609*, and *MMAA*) and 14 risky RNAs (*LINC00115*, *NCBP2-AS1*, *hsa-mir-34c*, *hsa-mir-363*, *hsa-mir-4709*, *hsa-mir-891a*, *ALOX15*, *ERVMER34–1*, *GRAP*, *KCP*, *NKAIN4*, *PADI1*, *SIX4*, and *SLC16A8*), which were used to establish the PS prediction model.

Kaplan-Meier curves showed that high risk group and low risk group in TCGA dataset divided by the PS prediction model had significantly different prognosis with a 5-year AUC of 0.951, which was also validated in GEO validation dataset with the 5-year AUC of 0.909. The prognosis prediction model constructed by independent prognostic clinical factors, including pathologic M and pathologic T, had a prognosis prediction efficiency with 5-year AUC of 0.712 and C-index of 0.680. The PS prediction model constructed by 17 optimum prognostic signature DERs had a better prognosis prediction efficiency with a 5-year AUC of 0.951 and C-index of 0.788. Huang et al. have developed a radiomics nomogram for preoperative prediction of lymph node metastasis with a C-index of 0.736, and validated in another cohort with a C-index of 0.778, suggesting this radiomics nomogram may be helpful for preoperative prediction of lymph node metastasis in colorectal cancer [30]. Another prognostic model composed of six significant prognostic factors (age, first-degree relative cancer history, differentiation grade, vessels/nerves invasion, TNM stage and *HALP*) has a 5-year AUC of 0.73 for patients with locally advanced colorectal cancer [31]. In our study, prognosis prediction model constructed by incorporating multi-RNAs with pathologic M and pathologic T has a 5-year AUC of 0.969 and a C-index of 0.812, suggesting this prognosis prediction model may have great value in COAD.

In the constructed ceRNA regulatory network, lncRNA *NCBP2-AS1* could interact with *hsa-miR-34c* and *hsa-miR-363*, and lncRNA *LINC00115* could interact with *hsa-miR-363* and *hsa-miR-4709*. *SIX4*, *GRAP*, *NKAIN4*, *MMAA*, and *ERVMER34–1* were regulated by *hsa-miR-4709*. It has been reported that lncRNA *LINC00115* might be a prognostic lncRNA in lung adenocarcinoma, and functions as a ceRNA by interacting with *hsa-miR-7* to regulate *FGF2* [32]. Zhang et al. have identified the differentially expressed lncRNAs related to cancer recurrence, and revealed that lncRNA *LINC00115* is one of the markedly up-regulated lncRNAs in cancer associated with disease-free survival [33]. In the study of Li et al., *hsa-miR-4709* was identified to be significantly up-regulated in colon cancer and high levels of *hsa-miR-4709* was associated with poor prognosis [34]. As one member of the sine oculis homeobox (SIX) homolog family, e *SIX4* have been found up-regulated in colorectal patients, which is significantly related to lymph node metastasis, advanced Tumor Node Metastasis (TNM) stages, and unfavorable prognosis [35]. Moreover, knocking down of *SIX4* could suppress colorectal cell metastasis via in-activation of the PI3K/Akt signaling pathway. Additionally, *SAM68* (Src-associated in mitosis, 68 kDa) has been reported overexpressed in colorectal cancer and higher expressions were related with poorer prognosis in colorectal cancer [36]. *SAM68* could interact with *GRAP* to active oncogenic pathways, such as epidermal growth factor and PI3K/Akt signaling pathways, thereby contributing to cancer progressing [37, 38]. Thus, lncRNA *LINC00115* identified as an optimum prognostic signature DERs in our study, may highly correlated with COAD recurrence via functioning as a ceRNA by interacting with *hsa-miR-4709* to regulate expressions of S*IX4*, *GRAP*, *NKAIN4*, *MMAA*, and *ERVMER34–1*. Moreover, in order to be further used in clinical, the prediction efficacy of the constructed prognosis prediction model, which incorporating multi-RNAs with pathologic M and pathologic T, should be considered to be validated by using a larger number of clinical samples.

## Conclusion

In conclusion, 562 DERs (42 lncRNAs, 36 miRNAs, and 484 mRNAs) were identified between 279 non-recurrent and 88 recurrent COAD samples downloaded from the TCGA. PS prediction model based on 17 optimum prognostic signature DERs was established, and validated in 65 COAD samples downloaded from GEO. Prognosis prediction model incorporating multi-RNAs with pathologic M and pathologic T has a 5-year AUC of 0.969 and a C-index of 0.812. The lncRNA *LINC00115* may highly correlated with COAD recurrence via functioning as a ceRNA by interacting with *hsa-miR-4709* to regulate expressions of *SIX4*, *GRAP*, *NKAIN4*, *MMAA*, and *ERVMER34-1*.

## Data Availability

The raw data were collected and analyzed by the Authors, and are not ready to share their data because the data have not been published.
